# Supplementary material for: Reconciling Mining with the Conservation of Cave Biodiversity: A Quantitative Baseline to Help Establish Conservation Priorities
Source: PLoS One. 2016 Dec 20;11(12):e0168348. doi: 10.1371/journal.pone.0168348 (PMC5173368; doi:10.1371/journal.pone.0168348)
Supplement: S1 Dataset — (ZIP) [file pone.0168348.s002.zip › Taxa/Serra Norte/SN_2013/N4E/Lista N4E-36.pdf]

CAVIDADE N4E-0036

Classe	Ordem	Fam/Outros	Gên/Outros	Espécie	S-U	U-S	Zon.
Arachnida	Amblypygi	Phrynidae	*Heterophrynus*	*longicornis*		X	P
Arachnida	Araneae	Corinnidae		sp.1	1		P
Arachnida	Araneae	Ctenidae	*Nothroctenus*	*marshi*	2		P
Arachnida	Araneae	Filistatidae		jovem	X	X	P
Arachnida	Araneae	Pholcidae	*Leptopholcus*	sp.1	X		P
Arachnida	Araneae	Pholcidae	*Mesabolivar*	aff. *cambridgei*	X		P
Arachnida	Araneae	Pholcidae	*Mesabolivar*	sp.		X	P
Arachnida	Araneae	Pholcidae	*Mesabolivar*	sp.1	X		P
Arachnida	Araneae	Salticidae		jovem		X	P
Arachnida	Araneae	Scytodidae	*Scytodes*	sp.		X	P
Arachnida	Araneae	Segestriidae		jovem		X	P
Arachnida	Araneae	Theridiosomatidae	*Plato*	sp.1	X	X	P
Diplopoda	Glomeridesmida	Glomeridesmidae		sp.		X	P
Entognatha	Diplura	Campodeidae		sp.1		X	P
Insecta	Blattodea	Polyphagidae		sp.1	1	X	P
Insecta	Diptera	Cecidomyiidae		sp.		X	P
Insecta	Diptera	Chironomidae		sp.	X		P
Insecta	Diptera	Psychodidae	Phlebotominae	sp.	X		P
Insecta	Diptera	Sciaridae		sp.	X		P
Insecta	Diptera	Tipulidae	Tipulinae	sp.	X		P
Insecta	Heteroptera	Reduviidae	Emesinae	sp.	1		P
Insecta	Heteroptera	Reduviidae	*Zelurus*	sp.	3	X	P
Insecta	Homoptera	Cixiidae		sp.		X	P
Insecta	Hymenoptera	Formicidae	*Camponotus*	*atriceps*		X	P
Insecta	Hymenoptera	Formicidae	*Camponotus*	sp.1	X		P
Insecta	Hymenoptera	Formicidae	*Camponotus*	sp.2	X		P
Insecta	Lepidoptera	Noctuoidea		sp.5	1		P
Insecta	Lepidoptera	Tineoidea		jovem		X	P
Insecta	Orthoptera	Phalangopsidae	*Paraclodes*	sp.	4		P
Insecta	Orthoptera	Phalangopsidae	*Phalangopsis*	sp.	2	X	P
Insecta	Thysanura	Nicoletiidae	Nicoletiinae	sp.1	X	X	P
Gastropoda	Pulmonata	Systrophiidae	*Happia*	sp.		X	P
Malacostraca	Isopoda	Philosciidae		sp.		X	P
Amphibia	Anura	Strabomantidae	*Pristimantis*	cf. *fenestratus*	3		P
Amphibia	Anura			sp.		1	P
Mammalia	Chiroptera	Emballonuridae	*Peropteryx*	*kappleri*	1	X	P
Mammalia	Rodentia	Cricetidae	*Rhipidomys*	sp.	1		P
Reptilia	Squamata	Sauria		sp.		1	P
